# Physical activity is associated with improved glycemic control but not with IL-6–glycemic interactions in type 2 diabetes: a cross-sectional study

**DOI:** 10.3389/fendo.2026.1837651

**Published:** 2026-06-05

**Authors:** Marwan Ismail, Mutaz Ibrahim Hassan, Amged Gaffer Mostafa, Ashgan A. Ahmed, Sara Mohammed Ali, Ellen Safadi, Ramprasad Muthukrishnan, Praveen Kumar Kandakurti, Asim Ahmed Elnour, Husham O. Elzein, Elryah I. Ali, Velilyaeva Alie Sabrievna, Ayman H. Alfeel

**Affiliations:** 1 Shendi University College of Medical Laboratory, Clinical Chemistry, Shendi, River Nile State, Sudan; 2 Department of Medical Laboratory Sciences, College of Health Sciences, Gulf Medical University, Ajman, United Arab Emirates; 3 Faculty of Medical and Health Sciences, Liwa University, Al Ain, United Arab Emirates; 4 Anaesthesia Technology Department, College of Health Sciences, Gulf Medical University, Ajman, United Arab Emirates; 5 Physiotherapy Division, College of Health Sciences, Gulf Medical University, Ajman, United Arab Emirates; 6 Program of Clinical Pharmacy, College of Pharmacy, Al Ain University, Abu Dhabi-United Arab Emirates (UAE). AAU Health and Biomedical Research Center, Al Ain University, Abu Dhabi, United Arab Emirates; 7 Department of Medical Laboratory Technology, College of Applied Medical Sciences, Northern Border University, Arar, Saudi Arabia; 8 Department of Medical Laboratory Technology, Faculty of Applied Medical Sciences, Northern Border University, Arar, Saudi Arabia; 9 Department of Psychiatry, Medical Psychology and Narcology, Samarkand State Medical University, Samarkand, Uzbekistan

**Keywords:** exercise modality, glycemic control, IL-6/IL-15 ratio, interleukin-6, myokine, physical activity, type 2 diabetes

## Abstract

**Background:**

Interleukin-6 (IL-6) exhibits both pro-inflammatory and exercise-induced myokine properties in type 2 diabetes mellitus (T2DM). Whether physical activity modifies the relationship between IL-6 and glycemic control remains unclear.

**Aim:**

To examine the associations of physical activity and circulating IL-6 with glycemic and metabolic markers in individuals with T2DM, and to test for interaction effects.

**Methods:**

A cross-sectional study with 185 T2DM participants (98 active, 87 sedentary) assessed circulating IL-6 levels alongside various glycemic and lipid markers. Multivariable regression with IL-6 × activity interaction terms was performed to test for effect modification.

**Results:**

Circulating IL-6 levels did not differ significantly between active and sedentary participants. Physical activity was associated with significantly lower HbA1c, fasting glucose, HOMA-IR, and fructosamine levels. In unadjusted analyses, IL-6 showed inverse correlations with glycemic markers in active individuals but not in sedentary participants. However, in multivariable models, IL-6 was not independently associated with glycemic outcomes, and no significant IL-6 × activity interaction effects were observed.

**Conclusions:**

Physical activity is associated with improved glycemic control in T2DM, whereas circulating IL-6 is not independently associated with metabolic outcomes and does not demonstrate activity-dependent interaction effects. Observed differences in unadjusted correlations should therefore be interpreted cautiously. These findings highlight the importance of accounting for confounding and interaction testing when evaluating cytokine–metabolic relationships. These findings are observational and hypothesis-generating and warrant confirmation in longitudinal studies.

## 1 Introduction

Type 2 diabetes mellitus (T2DM) is a chronic metabolic disorder characterized by progressive insulin resistance, β-cell dysfunction, and a sustained state of low-grade systemic inflammation often termed metaflammation (1, 2). The inflammatory component is not merely a downstream consequence of hyperglycemia but an active driver of disease progression. Expansion of visceral adipose tissue triggers macrophage infiltration and a phenotypic shift toward pro-inflammatory M1 polarization, resulting in elevated secretion of tumor necrosis factor-α (TNF-α), interleukin-1β (IL-1β), and chronically elevated interleukin-6 (IL-6) (2, 3). These cytokines activate serine kinase cascades most notably c-Jun N-terminal kinase (JNK) and inhibitor of κB kinase-β (IKK-β) which phosphorylate insulin receptor substrate-1 (IRS-1) at inhibitory serine residues, blunting downstream PI3K/Akt signaling and impairing GLUT-4–mediated glucose uptake in skeletal muscle and adipose tissue (2, 3). In parallel, NLRP3 inflammasome activation in pancreatic islets contributes to β-cell apoptosis and progressive secretory failure (2).

Counter-regulating this inflammatory environment is an endogenous anti-inflammatory axis that is functionally suppressed in sedentary T2DM but can be reactivated by habitual physical activity. Contracting skeletal muscle behaves as a secretory organ, releasing myokines that exert systemic anti-inflammatory effects through induction of interleukin-10 (IL-10) and interleukin-1 receptor antagonist (IL-1Ra), suppression of TNF-α production, and activation of AMP-activated protein kinase (AMPK) a central metabolic sensor that promotes glucose uptake, fatty-acid oxidation, and mitochondrial biogenesis while restraining mTORC1-driven anabolic stress (4–7). Among these myokines, IL-6 holds a paradoxical and clinically pivotal position: chronically elevated IL-6 of adipose-immune origin is a marker of metabolic deterioration, whereas acutely released muscle-derived IL-6 is a principal mediator of exercise’s anti-inflammatory and glucoregulatory benefits (3–5).

This functional duality has made IL-6 one of the most studied, yet least clinically resolved, biomarkers in the exercise-diabetes interface (2, 4). The prevailing concept of the “myokine hypothesis,” initially articulated by Pedersen and colleagues (8, 9), proposes that IL-6 released during sustained muscle contraction acts as an endocrine signal to coordinate multi-organ metabolic responses. In this context, muscle-derived IL-6 should convey favorable metabolic effects in physically active individuals effects that would be obscured or reversed in sedentary patients, where adipose tissue rather than skeletal muscle constitutes the dominant IL-6 source (3, 5, 10).

The metabolic effects of exercise in T2DM are modality-dependent. Aerobic training (e.g., brisk walking, cycling, swimming sustained at moderate intensity) predominantly enhances mitochondrial oxidative capacity, insulin sensitivity, and endothelial function, and elicits transient but pronounced muscle-derived IL-6 release proportional to contraction duration (10–12). Resistance training (progressive loading against external resistance) produces greater mechanical and eccentric stress, driving satellite-cell activation, hypertrophy, and a distinct myokine profile in which IL-6 elevations occur alongside sustained IL-15 induction (11, 13). Combined aerobic-plus-resistance regimens are increasingly recommended by international guidelines as the optimal modality for glycemic control in T2DM, on the premise that the two stimuli activate complementary signaling pathways (12, 14). The present sample reflects this clinical reality: among physically active participants, habitual exercise was categorized into aerobic-only, resistance-only, and combined modalities, while sedentary participants reported no engagement in structured exercise. Stratification by modality permits a preliminary examination of whether the metabolic associations of IL-6 differ according to the type of muscular work habitually performed, although the relatively small resistance and combined subgroups limit subgroup-level inference and warrant cautious interpretation.

Despite the theoretical clarity of this framework, human clinical data directly comparing circulating IL-6 levels and their metabolic correlates between active and sedentary T2DM patients remain limited (15, 16). Most published studies measure IL-6 either in acute exercise paradigms or in entirely sedentary diabetic populations, seldom stratifying participants by habitual physical activity status and examining the metabolic associations of IL-6 within each stratum separately (15). This gap is consequential: if the metabolic significance of IL-6 is conditioned by physical activity status, then interpreting IL-6 as a simple biomarker of inflammation or metabolic health without accounting for activity level introduces systematic error into both research and clinical interpretation (17).

The present study addresses this gap by examining IL-6 in a well-characterized, ethnically diverse cross-sectional cohort of 185 T2DM patients from the United Arab Emirates. We hypothesized that circulating IL-6 levels would not differ substantially between active and sedentary patients, but that the relationship between IL-6 and glycemic control markers might differ according to physical activity status. To formally test this, we examined IL-6 × activity interaction effects in multivariable models, in addition to descriptive stratified analyses.

## 2 Materials and methods

### 2.1 Study design and participants

This study was conducted as part of a larger cross-sectional investigation examining the relationships between physical activity, exercise modality, circulating myokine/adipokine profiles, and metabolic parameters in individuals with glycemic dysfunction attending outpatient clinics in Ajman, United Arab Emirates. From an initial recruitment pool, 192 T2DM patients were identified based on American Diabetes Association (ADA) diagnostic criteria. After applying pre-specified exclusion criteria including active insulin therapy (n=2) and clinical/laboratory suspicion of Latent Autoimmune Diabetes in Adults (LADA, n=5) a final T2DM cohort of 185 participants was included in the analysis. These exclusions were necessary because insulin-dependent pathophysiology and LADA represent mechanistically distinct conditions from typical T2DM and would introduce heterogeneity incompatible with the study’s comparisons.

The study cohort was demographically diverse, reflecting the multinational composition of the Ajman population. The largest group originated from the Indian subcontinent (41.1%), followed by Sudanese nationals (38.4%), participants from Arab countries including the UAE, Saudi Arabia, and Egypt (18.9%), and a small proportion from other regions (1.6%). This diversity enhances the external validity of findings beyond any single ethnic group.

This study was approved by the Gulf Medical University Institutional Review Board (Ref. No. IRB-COHS-STD-131- Dec-2024). All procedures were performed in accordance with institutional guidelines and the principles of the Declaration of Helsinki. Written informed consent was obtained from all participants.

#### 2.1.1 Inclusion and exclusion criteria

**2.1.1.1** Inclusion criteria were: (i) confirmed diagnosis of T2DM according to American Diabetes Association (ADA) criteria; (ii) stable pharmacological regimen for at least three months prior to enrollment; and (iii) ability to participate in standardized physical activity assessment.

**2.1.1.2** Exclusion criteria were: (i) type 1 diabetes mellitus or other endocrine or metabolic disorders that could alter the parameters under study (including untreated thyroid dysfunction and Cushing’s syndrome); (ii) use of insulin therapy or clinical/laboratory features suggestive of Latent Autoimmune Diabetes in Adults (LADA); (iii) history of cardiovascular events within the preceding six months; (iv) use of corticosteroids, immunosuppressants, or other agents known to influence systemic inflammation or cytokine profiles; (v) acute infection within four weeks of sampling; and (vi) pregnancy or active malignancy.

### 2.2 Physical activity assessment and stratification

Physical activity was assessed using the International Physical Activity Questionnaire (IPAQ), short form, which captures frequency, duration, and intensity of activity over the preceding seven days. Responses were converted into weekly MET-minutes following standard IPAQ scoring guidelines. Participants meeting the IPAQ threshold for moderate-to-high activity (≥600 MET-minutes/week with structured, regular exercise sessions) were classified as active (n=98, 53%), while those reporting fewer than 600 MET-minutes/week with no regular structured exercise were classified as sedentary (n=87, 47%).

Within the active group, exercise modality was further categorized based on the predominant training type reported: aerobic training (continuous moderate-intensity endurance activity such as walking, jogging, or cycling; n=70, 37.8%), resistance training (load-bearing muscular exercise; n=11, 5.9%), and combined aerobic and resistance training performed within the same training week (n=17, 9.2%). Sedentary participants reported no engagement in any structured, regular physical exercise.

### 2.3 Biochemical and biomarker measurements

Fasting venous blood specimens were obtained and analyzed biochemically using established, approved techniques. all blood samples were collected in the early morning (between 07:00 and 09:00) following an overnight fast of at least eight hours. Fasting plasma glucose (Ref. No. OSR6121) was measured using the enzymatic hexokinase method, while the lipid profile, including total cholesterol (Ref. No. OSR6116), triglycerides (Ref. No. OSR60118), and HDL cholesterol (Ref. No. A15625), was assessed through enzymatic colorimetric assays, all conducted on a Beckman Coulter AU/DxC 700 automated chemistry analyzer (Beckman Coulter, Brea, CA, USA). LDL cholesterol was determined via the Friedewald equation. Fructosamine (Ref. No. 08105677190; Roche Diagnostics, Mannheim, Germany) was quantified using the nitroblue tetrazolium (NBT) reduction technique on a Roche Cobas c analyzer. Glycated hemoglobin (HbA1c; Ref. No. B00389) was measured using turbidimetric immunoinhibition on the Beckman Coulter AU/DxC 700 platform. Fasting serum insulin (Ref. No. 33410) was quantified via a chemiluminescent immunoassay on a Beckman Coulter DxI 800 analyzer, and insulin resistance was assessed using the Homeostatic Model Assessment for Insulin Resistance (HOMA-IR), calculated as: fasting insulin (μU/mL) × fasting glucose (mg/dL)/405. The circulating quantities of interleukin-6 (IL-6; Cat. No. ELK1156) and interleukin-15 (IL-15; Cat. No. ELK1146) were quantified using commercially available sandwich enzyme-linked immunosorbent assay (ELISA) kits (ELK Biotechnology, Wuhan, China), in accordance with the manufacturer’s guidelines. Optical density was measured at 450 nm with a TECAN Sunrise microplate reader, and analyte concentrations were derived from four-parameter logistic standard curves. All tests were conducted following the relevant manufacturer’s procedures; comprehensive data, including supplier names, catalogue numbers, and assay performance parameters.

All ELISA washing procedures were conducted using a semi-automated microplate washer (Tecan HydroFlex, Serial No. 12050000331; Tecan Group Ltd., Switzerland) to maintain consistency and reduce background signal. Absorbance was quantified at 450 nm using a TECAN Sunrise Basic microplate reader (Serial No. 1205000443; Tecan Group Ltd., Switzerland). Duplicate OD measurements were averaged, adjusted for blanks, and analyzed using GainData (Arigo Biolaboratories), a web-based application using four- or five-parameter logistic regression for standard curve fitting and sample quantification. Final concentrations were determined by using the relevant dilution factor.

### 2.4 Sampe size and statistical analysis

#### 2.4.1 Sampe size

Sample size was calculated using GPower 3.1.9.7. For Pearson correlation analysis, assuming a medium effect size (r = 0.3), a two-tailed significance level of α = 0.05, and statistical power of 80%, a minimum of 85 participants was required. To accommodate stratified analyses by physical activity status, account for measurement variability, and ensure robust subgroup comparisons, a target of 100–120 participants was considered appropriate. The final enrolled sample of 185 participants exceeded this target, providing adequate power to detect moderate correlations (|r| ≥ 0.28) within each activity stratum (active n=98; sedentary n=87). Modality-stratified comparisons within the active group remained underpowered given the small resistance (n=11) and combined training (n=17) subgroups and are therefore considered exploratory and hypothesis-generating.

#### 2.4.2 Statistical analysis

Given the non-normal distribution of most biomarker variables, non-parametric tests were applied throughout. Between-group comparisons of continuous variables used the Mann–Whitney U test (active vs. sedentary). Comparisons across three exercise modality groups used the Kruskal–Wallis test. Given the relatively small sample sizes in the resistance (n=11) and combined training (n=17) subgroups, modality-stratified comparisons were considered exploratory and hypothesis-generating. Correlational analyses between L-6 and glycemic/lipid markers, and between the IL-6/IL-15 ratio and metabolic parameters, were conducted using Spearman’s rank correlation coefficient, computed separately within active and sedentary strata. A p-value threshold of 0.05 was applied for statistical significance. All statistical analyses were conducted using SPSS version 30.

Multivariable linear regression analyses were performed to evaluate the independence of the observed associations after adjustment for potential confounders. IL-6 and the IL-6/IL-15 ratio were modeled as dependent variables, with age, BMI, sex, and medication status entered as covariates. Regression coefficients (β) with 95% confidence intervals and corresponding *p*-values were reported, and statistical significance was set at *p* < 0.05.

To formally test whether the relationship between IL-6 and glycemic markers differed by physical activity status, we constructed multivariable linear regression models with each glycemic outcome (fasting glucose, fasting insulin, HOMA-IR, HbA1c and Fructosamine) as the dependent variable. Predictors included standardized IL-6, physical activity status (active vs sedentary), and an IL-6 × activity interaction term, with adjustment for age, BMI, and sex. Robust (HC3) standard errors were used to account for heteroscedasticity. The interaction term tests whether the IL-6 slope on the glycemic outcome differs between activity groups. False discovery rate (FDR) correction was applied across the five outcomes for each predictor.

## 3 Results

### 3.1 Participant characteristics

The T2DM cohort (n=185) was predominantly male (67%, n=124), with the majority falling in the 45–59 year age category (47.6%, n=88). Body mass index (BMI) distribution revealed that 38.9% were overweight and 32.9% had obesity of varying classes. The most common pharmacological treatment was metformin monotherapy, with a subset receiving combination oral therapy. Medication distributions were broadly comparable between active and sedentary groups, minimizing pharmacological confounding of biomarker comparisons (**Table 1**).

**Table 1:** Demographic and anthropometric characteristics of the DM2 Study Population (n = 185).

Variable	groups	N	%
Age group (WHO)	<45	53	28.6
	45–59	88	47.6
	>60	44	23.8
Gender	Female	61	33
	Male	124	67
BMI	Normal	48	25.9
	Underweight	4	2.2
	Obesity Class I	40	21.6
	Obesity Class II	15	8.1
	Obesity Class III	6	3.2
	Overweight	72	38.9
Physical Activity Status	Active	98	53
	Sedentary	87	47
Exercise Subgroup	Aerobic	70	37.8
	Combined	17	9.2
	Resistance	11	5.9

Percentages are expressed relative to the total sample (N = 185). Exercise subgroup applies only to physically active participants (n = 98).

### 3.2 Circulating IL-6 levels: active vs. sedentary

Median circulating IL-6 concentrations were 7.1 pg/mL (IQR 4.86–9.42) in physically active participants and 7.81 pg/mL (IQR 5.12–12.16) in sedentary participants. This difference was not statistically significant (Mann–Whitney U test, p = 0.175), indicating that resting IL-6 levels did not differ between groups in this cross-sectional cohort of T2DM patients. The IQR widths suggest modestly greater variability in the sedentary group, consistent with more heterogeneous inflammatory backgrounds in physically inactive individuals, but this pattern did not translate into a statistically detectable median difference (**Table 2**).

**Table 2:** Comparison of IL6 levels and other cardiometabolic biomarkers between active and sedentary DM2 patients groups.

Variable	Median (IQR)		P Value
	Active (n = 98	Sedentary (n = 87)	
Weight (kg)	80 (68.5–85.0)	82 (72.1–96.5)	0.068
BMI (kg/m²)	26.02 (23.7–30.28)	28.7 (26.45–33.45)	<0.001
A1C (%)	6.84 (6.49–8.04)	8.07 (7.14–9.61)	<0.001
Fasting Glucose (mg/dL)	127 (118.7–153.2)	150 (129.5–200.0)	<0.001
Fasting Insulin (μU/mL)	8.88 (5.85–13.82)	11.17 (7.78–19.24)	0.008
HOMA-IR	2.95 (1.9–4.7)	4.1 (2.7–8.05)	<0.001
Fructosamine (μmol/L)	301 (269.5–369.75)	362 (319.25–405.75)	<0.001
Total Cholesterol (mg/dL)	179 (149.0–211.0)	184 (162.0–217.5)	0.371
Triglycerides (mg/dL)	139 (98.0–182.75)	135 (97.5–186.0)	0.985
LDL (mg/dL)	107.4 (82.0–131.5)	113 (94.2–133.0)	0.556
HDL (mg/dL)	45 (38.0–53.0)	46 (40.0–52.5)	0.252
IL-6 (pg/mL)	7.1 (4.86–9.42)	7.81 (5.12–12.16)	0.175
IL-15 (pg/mL)	104.95 (72.4–213.19)	111.93 (73.97–173.04)	0.794

**Table 2** presents a comprehensive comparison of IL6 levels and other cardiometabolic biomarkers between active and sedentary groups DM2 patients data are presented as medians with interquartile ranges (IQR) to account for non-normal distributions. Overall group differences were assessed using the Mann–Whitney U tests. P.value less than 0.05 is considered significant.

### 3.3 IL-6 by exercise modality

Among the 98 active participants, IL-6 levels varied across exercise modalities but did not reach statistical significance (Kruskal–Wallis, p=0.128).The median IL-6 levels were 6.86 pg/mL (IQR 4.46–8.68) in the aerobic group, 9.42 pg/mL (IQR 5.62–11.40) in the resistance training group, and 8.62 pg/mL (IQR 5.88–9.42) in the combined training group. The higher IL-6 levels in the resistance and combined groups compared to the aerobic group align with the increased mechanical loading and muscle damage characteristic of these modalities. However, the difference between groups did not reach statistical significance, likely due to the small subgroup sizes (n=11 and n=17, respectively) (**Table 3**).

**Table 3:** Comparison of IL6 levels across three distinct exercise modality groups in physically active type 2 diabetes patients (n=98).

Parameter	*Median (interquartile range)*			p-value
Exercise Modality	Aerobic (n = 70)	Resistance (n = 11)	Combined (n = 17)	
IL-6 (pg/ml)	6.861 (4.462–8.682)	9.421 (5.616–11.402)	8.624 (5.879–9.421)	0.128

**Table 3** presents a comprehensive comparison of IL6 levels across three distinct exercise modality groups in physically active type 2 diabetes patients (n=98): aerobic training (n=70), resistance training (n=11), and combined modality training (n=17). Data are presented as medians with interquartile ranges (IQR) to account for non-normal distributions. Overall group differences were assessed using the Kruskal–Wallis test. P.value less than 0.05 is considered significant.

### 3.4 IL-6 correlations with glycemic markers by activity status

Despite the absence of a between-group level difference, the correlational pattern of IL-6 with glycemic markers differed between active and sedentary strata in unadjusted Spearman analyses.

In physically active participants, IL-6 demonstrated significant negative correlations with all three principal glycemic markers: HbA1c (r=−0.29, p=0.003), fasting glucose (r=−0.219, p=0.030), and fructosamine (r=−0.398, p<0.001). This pattern indicates that among active T2DM patients, higher circulating IL-6 is associated with better glycemic control across short-, intermediate-, and long-term glucose metrics simultaneously.

In sedentary participants, by contrast, none of these unadjusted correlations were statistically significant: IL-6 showed no meaningful association with HbA1c, fasting glucose, or fructosamine in this group.

Neither group showed significant correlations between IL-6 and HOMA-IR or fasting insulin, suggesting that any apparent IL-6–glycemia associations in active participants are not captured by surrogate insulin resistance indices (**Table 4**).

**Table 4:** IL6 levels correlations with glycemic markers in active (n = 98) versus sedentary (n = 87) patients.

Myokine	Glycemic marker	r	P.value	Direction	Significance	r	P.value	Direction	Significance
		Active (n = 98)				Sedentary (n = 87)			
IL6 (pg/ml)	A1C (%)	-0.29	0.003	negative	significant	0.071	0.515	positive	non-significant
	Fasting Glucose (mg/dl)	-0.219	0.030	negative	significant	0.057	0.599	positive	non-significant
	Fasting Insulin (uU/mL)	0.029	0.778	positive	non-significant	-0.061	0.573	negative	non-significant
	Fructosamine umol/L	-0.398	< 0.001	negative	significant	-0.13	0.233	negative	non-significant
	HOMA IR	-0.091	0.371	negative	non-significant	0.015	0.892	positive	non-significant

**Table 4** Spearman correlation coefficients (r) between circulating IL6 levels and glycemic markers are presented separately for active (n = 98) and sedentary (n = 87) patients with type 2 diabetes. The table shows r and corresponding p value in both groups, together with the direction of association (positive or negative) and whether the correlation reached statistical significance (p < 0.05).

These stratified correlations are descriptive findings from unadjusted analyses; their statistical robustness is formally evaluated in the multivariable interaction analysis presented in Section 3.7.

### 3.5 The IL-6/IL-15 composite ratio and glycemic correlations

The IL-6/IL-15 ratio analysis yielded the most notable outcome among all the biomarker correlation panels. In active participants, this ratio demonstrated strong, significant negative correlations with fructosamine (r=−0.539, p<0.001), HbA1c (r=−0.352, p<0.001), and fasting glucose (r=−0.296, p=0.003). Out of all the individual and composite biomarkers analyzed, the fructosamine correlation was the strongest.

In sedentary patients, the ratio showed only a single significant association, with fructosamine (r=−0.216, p=0.046), and no significant correlations with fasting glucose or HbA1c.

In unadjusted analyses, the relative balance between IL-6 and IL-15 two myokines with partially overlapping but distinct exercise-responsive roles showed stronger correlations with glycemic markers in physically active individuals than either cytokine alone, although these associations were not subsequently confirmed in adjusted models (**Table 5**, Section 3.6).

**Table 5:** Correlations of IL6_to_IL15 ratios with glycemic control and lipid profile in active (n = 98) versus sedentary (n = 87) patients.

Composite ratio	Marker	r	P.value	Direction	Significance	r	P.value		Direction	Significance
		Active (n = 98)				Sedentary (n = 87)				
IL6 to IL15	Fasting Glucose (mg/dl)	-0.296	0.003	negative	significant	0.02		0.856	positive	non-significant
	Fasting Insulin (uU/mL)	-0.077	0.452	negative	non-significant	-0.076		0.485	negative	non-significant
	HOMA IR	-0.194	0.055	negative	non-significant	-0.042		0.698	negative	non-significant
	A1C (%)	-0.352	0.000	negative	significant	0.167		0.123	positive	non-significant
	Fructosamine umol/L	-0.539	< 0.001	negative	significant	-0.216		0.046	negative	significant
	Cholesterol (mg/dl)	-0.17	0.093	negative	non-significant	0.094		0.387	positive	non-significant
	Triglyceride (mg/dl)	0.063	0.539	positive	non-significant	-0.086		0.428	negative	non-significant
	LDL (mg/dl)	-0.138	0.174	negative	non-significant	0.171		0.114	positive	non-significant
	HDL (mg/dl)	-0.145	0.155	negative	non-significant	-0.061		0.573	negative	non-significant

Spearman correlation coefficients (r) between composite biomarker ratios IL-6-to-IL-15) and indices of glycemic control (fasting glucose, fasting insulin, HOMA-IR, A1C, Fructosamine) and lipid profile (total cholesterol, triglycerides, LDL, HDL) are presented separately for active (n = 98) and sedentary (n = 87) patients with type 2 diabetes. For each ratio–marker pair, r and p values, direction of association, and statistical significance (p < 0.05) are shown, allowing direct comparison between activity groups.

### 3.6 Multivariable regression analysis

To assess whether the observed associations remained robust after accounting for potential confounders, multivariable linear regression models were constructed using IL-6 and the IL-6/IL-15 ratio as dependent variables, with age, BMI, sex, and medication status entered as covariates (**Table 6**).

**Table 6:** Multivariable linear regression analysis of IL-6 and IL-6/IL-15 ratio as outcomes adjusted for age, BMI, sex, and medication status (n = 185).

Predictor	β	Robust SE	95% CI	p-value
A. Outcome: IL-6 (log1p) Model summary: R² = 0.023; Adjusted R² = −0.004:				
Age	-0.0051	0.0066	-0.0180 to 0.0078	0.442
BMI	0.0172	0.0125	-0.0073 to 0.0418	0.168
Female vs Male	-0.0314	0.1772	-0.3788 to 0.3160	0.859
Other/combination medication vs metformin only	-0.1512	0.2158	-0.5742 to 0.2717	0.483
B. Outcome: IL-6/IL-15 Ratio (log1p) Model summary: R² = 0.009; Adjusted R² = −0.019				
Age	0.0006	0.0022	-0.0037 to 0.0048	0.801
BMI	0.0038	0.0052	-0.0064 to 0.0139	0.465
Female vs Male	-0.0011	0.1772	-0.1355 to 0.1333	0.988
Other/combination medication vs metformin only	-0.0375	0.0766	-0.1877 to 0.1127	0.625

β, standardized regression coefficient; SE, standard error; CI, confidence interval.

All models adjusted for age, BMI, sex, and medication status. Statistically significant values (p < 0.05).

For log-transformed IL-6, none of the examined predictors reached statistical significance: age (β = −0.0051, p = 0.442), BMI (β = 0.0172, p = 0.168), sex (β = −0.0314, p = 0.859), and medication status (β = −0.1512, p = 0.483). The overall model accounted for only a small fraction of the variance in IL-6 levels (R² = 0.023).

A comparable pattern emerged for the log-transformed IL-6/IL-15 ratio, with no covariate showing a significant independent contribution (all p > 0.05) and the model explaining minimal variance (R² = 0.009).

Taken together, these results suggest that age, BMI, sex, and medication status do not meaningfully account for inter-individual variability in IL-6 concentrations or in the IL-6/IL-15 ratio. Accordingly, the univariate associations identified earlier did not persist after multivariable adjustment, implying that other unmeasured biological or behavioral factors may underlie the observed cytokine variation in this cohort.

### 3.7 Interaction analysis: IL-6, physical activity, and glycemic outcomes

To formally test whether the relationship between IL-6 and glycemic markers differed by physical activity status, multivariable models with IL-6 × activity interaction terms were constructed (**Table 7**). Physical activity was independently associated with markedly lower glycemic markers across multiple outcomes after adjustment for age, BMI, and sex: fasting glucose (β = −31.0, p = 0.001), HOMA-IR (β = −2.09, p = 0.005), HbA1c (β = −0.98, p < 0.001), and fructosamine (β = −43.4, p = 0.003). These activity effects survived FDR correction.

**Table 7:** Multivariable linear regression models examining associations of IL-6, physical activity, and IL-6 × activity interaction with glycemic outcomes in T2DM (n = 185).

Outcome	Predictor	β	SE	95% CI	p-value
Fasting Glucose (mg/dL)	IL-6 (per SD), sedentary	-5.41	3.85	-12.96 to 2.15	0.161
	Active vs sedentary	-31.01	9.46	-49.56 to -12.46	0.001
	IL-6 × activity	-1.35	4.49	-10.15 to 7.44	0.763
Fasting Insulin (µU/mL)	IL-6 (per SD), sedentary	-1.02	0.69	-2.37 to 0.33	0.139
	Active vs sedentary	-3.41	1.76	-6.86 to 0.05	0.053
	IL-6 × activity	0.98	0.90	-0.78 to 2.74	0.273
HOMA-IR	IL-6 (per SD), sedentary	-0.53	0.32	-1.15 to 0.09	0.094
	Active vs sedentary	-2.09	0.75	-3.56 to -0.62	0.005
	IL-6 × activity	0.37	0.37	-0.37 to 1.10	0.326
HbA1c (%)	IL-6 (per SD), sedentary	-0.10	0.11	-0.31 to 0.11	0.351
	Active vs sedentary	-0.98	0.28	-1.53 to -0.43	<0.001
	IL-6 × activity	-0.21	0.15	-0.50 to 0.08	0.149
Fructosamine (µmol/L)	IL-6 (per SD), sedentary	-18.26	8.45	-34.81 to -1.70	0.031
	Active vs sedentary	-43.40	14.59	-71.99 to -14.80	0.003
	IL-6 × activity	-12.61	10.78	-33.74 to 8.52	0.242

IL-6, interleukin-6; SE, standard error; CI, confidence interval; HOMA-IR, homeostatic model assessment of insulin resistance. All models include IL-6 (standardized), physical activity status (active vs sedentary), and an IL-6 × activity interaction term. Models are adjusted for age, BMI, and sex. Robust (HC3) standard errors were used.

Among sedentary participants, IL-6 showed a significant inverse association with fructosamine (β = −18.3, p = 0.031), although this did not survive FDR correction; no other IL-6 main effects in the sedentary stratum reached significance. Crucially, none of the IL-6 × activity interaction terms reached statistical significance for any glycemic outcome (all p ≥ 0.083, all FDR-adjusted p > 0.45). These findings indicate that, after adjustment for confounders, physical activity is robustly associated with better glycemic control, but the magnitude of the IL-6–glycemic marker relationship does not differ significantly between active and sedentary patients.

## 4 Discussion

This study set out to examine the interplay between physical activity, circulating IL-6, and metabolic indices in patients with T2DM. The findings show that resting IL-6 concentrations did not differ significantly between active and sedentary participants, and that physical activity itself independently of IL-6 was robustly associated with better glycemic control. Stratified analyses suggested differential IL-6–glycemia correlations by activity status, but a formal interaction test did not confirm effect modification (**Table 7**). The dissociation between IL-6 concentration and metabolic function therefore remains a hypothesis that warrants direct testing in longitudinal and tissue-resolved studies, rather than a finding established by the present data (4, 5).

The absence of a significant IL-6 difference between active and sedentary groups is, in retrospect, biologically expected. Resting IL-6 concentrations after habitual exercise training represent a complex resultant of at least three competing processes: reduced adipose-derived inflammatory IL-6 (reflecting lower visceral fat in trained individuals), potentially increased basal myokine IL-6 output from conditioned muscle, and altered hepatic clearance (4, 5, 18). In a cross-sectional design with a relatively modest BMI difference between groups (26.02 vs. 28.7 kg/m², p<0.001), these opposing forces plausibly cancel to produce overlapping resting concentrations. This is consistent with meta-analytic data showing that exercise training produces only modest reductions in resting IL-6 in established T2DM, in contrast to more pronounced effects in healthy or pre-diabetic populations (15, 16).

What is not cancelled, in unadjusted analyses, is the direction and significance of IL-6’s correlations with glycemic control among active participants. However, when formally tested in multivariable models with an IL-6 × activity interaction term (**Table 7**), this apparent activity-dependent pattern did not reach statistical significance. The differential correlations observed in stratified Spearman analyses therefore likely reflect within-group variation rather than true effect modification, and the ‘myokine arm’ interpretation should be regarded as a hypothesis our data could not confirm. Notwithstanding this null interaction result, the literature offers a plausible mechanistic interpretation: contraction-induced IL-6 acts through AMPK-mediated pathways to stimulate GLUT-4 translocation, enhance peripheral glucose uptake, and suppress hepatic glucose output (6, 18), and may also stimulate GLP-1 secretion from intestinal L-cells, contributing to improved postprandial glycemic control (19–21). These pathways were not directly assessed in the present study and were not supported by our interaction analysis; we frame this interpretation as a hypothesis derived from prior experimental literature rather than a mechanism demonstrated by our cross-sectional findings.

IL-6 has been shown to interact with the AMPK–mTOR signaling axis, with AMPK activation generally restraining mTORC1 activity and shifting cellular metabolism toward catabolic, glucose-disposing pathways (22). Exercise itself is a well-recognized modulator of this balance: acute contraction transiently activates mTORC1 to promote muscle protein synthesis, while chronic training conditions favor AMPK-dominant signaling that supports glucose uptake and oxidative metabolism (23). mTORC1 signaling has also been implicated in pancreatic β-cell function and metabolic disease through phosphorylation of key transcription factors such as PDX1 (24). It is therefore conceivable that the divergent IL-6–glycemia associations observed between active and sedentary participants in our study reflect, at least in part, an exercise-conditioned shift in the AMPK–mTOR balance favoring catabolic, glucose-disposing signaling in active individuals and a pro-inflammatory, mTOR-skewed signaling environment in sedentary patients. This interpretation, while consistent with current mechanistic literature, was not directly tested in the present study and is presented as a hypothesis warranting future experimental validation.

Tissue origin of circulating IL-6 skeletal muscle versus visceral adipose tissue and activated macrophages has been proposed to determine its metabolic consequences (3, 5, 17). The present cross-sectional design could not distinguish IL-6 sources, and the IL-6 × activity interaction was not statistically significant for any glycemic outcome (**Table 7**). Whether tissue-of-origin underlies any activity-dependent IL-6 metabolic signaling therefore remains an open question requiring tissue-resolved investigation (4, 25).

The IL-6/IL-15 ratio findings deserve specific commentary. IL-15, like IL-6, is a muscle-derived myokine with exercise-induced characteristics, including roles in mitochondrial biogenesis, skeletal muscle hypertrophy, and anti-adipogenic effects (13, 26). The two cytokines are not functionally redundant: IL-15 shows relatively sustained elevations with chronic training, whereas IL-6 is more acutely responsive to each exercise bout (13). A higher IL-6/IL-15 ratio in the active group may therefore reflect the acute exercise component of recent activity, while a lower ratio might indicate a more chronically trained, IL-15-dominant myokine environment. The near-universal significance of this ratio’s correlations with glycemic markers in active patients, and its near-complete absence in sedentary patients, was observed in unadjusted analyses only (8, 26). As with IL-6 itself, the IL-6/IL-15 ratio was not independently associated with metabolic parameters in multivariable models (**Table 6**). Although the unadjusted fructosamine correlation (r=−0.539) is notable for its magnitude, the multivariable findings caution against interpreting this descriptive pattern as evidence of activity-dependent myokine signaling.

The exercise modality data, while underpowered for IL-6 specifically, raise an interesting secondary observation. Resistance training was associated with numerically higher IL-6 compared to aerobic training (9.42 vs. 6.86 pg/mL), consistent with the well-documented higher acute IL-6 response to eccentric and resistance exercise compared to sustained aerobic work (10, 11). The combined training group showed intermediate values. These modality-specific differences were not statistically significant, likely due to the small resistance and combined subgroup sizes. Future studies with larger resistance training cohorts are needed to determine whether IL-6 modality differences are clinically meaningful in T2DM (2, 27).

A key finding of this study is that the associations observed in univariate analyses were not maintained after adjustment for confounding variables. Multivariable regression analysis demonstrated that neither IL-6 nor the IL-6/IL-15 ratio was independently associated with age, BMI, sex, or medication status, and both models showed very low explanatory power.

Furthermore, formal IL-6 × activity interaction testing (**Table 7**) showed that the apparent activity-dependent IL-6–glycemia pattern observed in stratified Spearman analyses was not statistically supported. Physical activity itself, however, was robustly and independently associated with lower fasting glucose, HbA1c, HOMA-IR and Fructosamine across multivariable models, with effects surviving FDR correction. Together, these results reposition physical activity, rather than IL-6 modulation, as the dominant statistically supported correlate of glycemic control in this cohort.

## 5 Limitations

Several limitations merit acknowledgment and have meaningful implications for the interpretation of these findings. First, the cross-sectional design precludes causal inference; longitudinal or interventional data would be required to confirm whether exercise training actively reorients IL-6 metabolic signaling rather than co-occurring with favorable glycemia through shared confounders. Second, although self-reported physical activity classification is standard in large epidemiological studies, it did not capture exercise duration, intensity, or timing relative to blood sampling each of which materially influences IL-6 kinetics (15) and direct comparison of exercise duration across active participants was therefore not feasible. Third, the relatively small number of participants engaged in resistance and combined exercise modalities limited subgroup analyses and reduced the statistical power to detect modality-specific effects. Fourth, circulating IL-6 fluctuates according to sampling time and transient individual conditions (e.g., recent meals, sleep, acute stress), and peripheral measurements cannot disentangle the relative contributions of pro-inflammatory (immune/adipose-derived) versus anti-inflammatory (muscle-derived myokine) sources; tissue-specific or receptor-level data would considerably strengthen mechanistic interpretation (5, 17). Finally, the broader anti-inflammatory response involves additional mediators including IL-10 and its interactions with IL-1 and TNF-α which were not measured in the present study and may modulate the net metabolic impact of IL-6. The cohort was also drawn from a single clinical region in the UAE and, while ethnically diverse, may not be fully representative of global T2DM populations. Collectively, these limitations temper the strength of the conclusions and underscore the need for longitudinal, multi-cytokine, and tissue-resolved investigations to clarify the causal architecture linking physical activity, IL-6 signaling, and glycemic control.

## 6 Conclusions

This cross-sectional study suggests that physical activity is robustly and independently associated with better glycemic control across multiple markers (fasting glucose, HbA1c, HOMA-IR, and fructosamine) in T2DM, after adjustment for age, BMI, and sex (12, 14). While unadjusted analyses showed inverse correlations between IL-6 and glycemic markers in physically active participants and a similar pattern for the IL-6/IL-15 ratio (13, 26) neither IL-6 nor the ratio remained independently associated with metabolic parameters after multivariable adjustment, and formal IL-6 × activity interaction testing did not support effect modification by physical activity status (15, 17). These findings indicate that, in this cohort, the apparent activity-dependent IL-6 correlations seen in stratified analyses likely reflect statistical artifacts of stratification rather than true biological effect modification. From a clinical and research perspective, the results reinforce physical activity itself as the principal correlate of glycemic control in T2DM (12, 14) and underscore the importance of formal interaction testing when evaluating cytokine–metabolic relationships across activity strata. The hypothesis that exercise functionally reprograms IL-6 signaling remains plausible based on prior experimental literature (4–6, 18), but was not statistically supported by the present cross-sectional data and warrants direct testing in longitudinal and mechanistic studies (16).

## Data Availability

The raw data supporting the conclusions of this article will be made available by the authors, without undue reservation.

## References

[1] American Diabetes Association Professional Practice Committee . Standards of care in diabetes—2024. Diabetes Care. (2024) 47:S1–S321. 38078587

[2] Chen ZT Weng ZX Lin JD Meng ZX . Myokines: metabolic regulation in obesity and type 2 diabetes. Life Metab. (2024) 3:loae006. doi: 10.1093/lifemeta/loae006. PMID: 39872377 PMC11749576

[3] Donath MY Shoelson SE . Type 2 diabetes as an inflammatory disease. Nat Rev Immunol. (2011) 11:98–107. doi: 10.1038/nri2925. PMID: 21233852

[4] Kistner TM Pedersen BK Lieberman DE . Interleukin 6 as an energy allocator in muscle tissue. Nat Metab. (2022) 4:170–9. doi: 10.1038/s42255-022-00538-4. PMID: 35210610

[5] Wang J Chen J . Functional role of skeletal muscle-derived interleukin-6 and its effects on lipid metabolism. Front Physiol. (2023) 14:1110926. doi: 10.3389/fphys.2023.1110926. PMID: 37555019 PMC10405179

[6] Carey AL Steinberg GR Macaulay SL Thomas WG Holmes AG Rodnick KJ . Interleukin-6 increases insulin-stimulated glucose disposal in humans and glucose uptake and fatty acid oxidation *in vitro* via AMP-activated protein kinase. Diabetes. (2006) 55:2688–97. doi: 10.2337/db05-1404. PMID: 17003332

[7] Petersen AMW Pedersen BK . The anti-inflammatory effect of exercise. J Appl Physiol. (2005) 98:1154–62. doi: 10.1152/japplphysiol.00164.2004. PMID: 15772055

[8] Severinsen MCK Pedersen BK . Muscle–organ crosstalk: the emerging roles of myokines. Endocr Rev. (2020) 41:594–609. doi: 10.1210/endrev/bnaa016. PMID: 32393961 PMC7288608

[9] Pedersen BK Febbraio MA . Muscles, exercise and obesity: skeletal muscle as a secretory organ. Nat Rev Endocrinol. (2012) 8:457–65. doi: 10.1038/nrendo.2012.49. PMID: 22473333

[10] Brandt C Pedersen BK . The role of exercise-induced myokines in muscle homeostasis and the defense against chronic diseases. J BioMed Biotechnol. (2010) 2010:520258. doi: 10.1155/2010/520258. PMID: 20224659 PMC2836182

[11] Serrano AL Baeza-Raja B Perdiguero E Jardí M Muñoz-Cánoves P . Interleukin-6 is an essential regulator of satellite cell-mediated skeletal muscle hypertrophy. Cell Metab. (2008) 7:33–44. doi: 10.1016/j.cmet.2007.11.011. PMID: 18177723

[12] Pan Y Wang P Yue C Liu C . Effect of nine different exercise interventions on insulin sensitivity in diabetic patients: a systematic review and mesh meta-analysis. Front Endocrinol (Lausanne). (2025) 16:1409474. doi: 10.3389/fendo.2025.1409474 40951419 PMC12424434

[13] Quinn LS Anderson BG Strait-Bodey L Stroud AM Argilés JM . Oversecretion of interleukin-15 from skeletal muscle reduces adiposity. Am J Physiol Endocrinol Metab. (2009) 296:E191–202. doi: 10.1152/ajpendo.90506.2008. PMID: 19001550 PMC2636988

[14] Mendes R Sousa N Almeida A Subtil P Guedes-Marques F Reis VM . Exercise prescription for patients with type 2 diabetes—a synthesis of international recommendations: narrative review. Br J Sports Med. (2016) 50:1379–81. doi: 10.1136/bjsports-2015-094895. PMID: 26719499

[15] Hamasaki H . Effects of exercise on circulating muscle-related cytokines in adults with type 2 diabetes and/or obesity. Curr Diabetes Rev. (2023) 19:e150622206289. doi: 10.2174/1573399819666221212145712. PMID: 36515029

[16] García-Hermoso A Ramírez-Vélez R Díez J González A Izquierdo M . Exercise training-induced changes in exerkine concentrations may be relevant to the metabolic control of type 2 diabetes mellitus patients: a systematic review and meta-analysis of randomized controlled trials. J Sport Health Sci. (2023) 12:147–57. doi: 10.1016/j.jshs.2022.11.003. PMID: 36351545 PMC10105032

[17] Jiang LQ De Castro Barbosa T Massart J Deshmukh AS Löfgren L Björnholm M . Altered response of skeletal muscle to IL-6 in type 2 diabetic patients. Diabetes. (2013) 62:355–61. doi: 10.2337/db11-1790. PMID: 23086036 PMC3554367

[18] de Melo Madureira ÁN de Oliveira JRS de Menezes Lima VL . The role of IL-6 released during exercise to insulin sensitivity and muscle hypertrophy. Mini-Rev Med Chem. (2022) 22:2419–28. doi: 10.2174/1389557522666220309161245. PMID: 35264090

[19] Ellingsgaard H Hauselmann I Schuler B Habib AM Baggio LL Meier DT . Interleukin-6 enhances insulin secretion by increasing glucagon-like peptide-1 secretion from L cells and alpha cells. Nat Med. (2011) 17:1481–9. doi: 10.1038/nm.2513. PMID: 22037645 PMC4286294

[20] Ellingsgaard H Seelig E Timper K Coslovsky M Soederlund L Lyngbaek MP . GLP-1 secretion is regulated by IL-6 signalling: a randomised, placebo-controlled study. Diabetologia. (2020) 63:362–73. doi: 10.1007/s00125-019-05045-y. PMID: 31796986

[21] Kahles F Meyer C Möllmann J Diebold S Findeisen HM Lebherz C . GLP-1 secretion is increased by inflammatory stimuli in an IL-6-dependent manner, leading to hyperinsulinemia and blood glucose lowering. Diabetes. (2014) 63:3221–9. doi: 10.2337/db14-0100. PMID: 24947356

[22] Ruderman NB Keller C Richard AM Saha AK Luo Z Xiang X . Interleukin-6 regulation of AMP-activated protein kinase: potential role in the systemic response to exercise and prevention of the metabolic syndrome. Diabetes. (2006) 55:S48–54. doi: 10.2337/db06-s007. PMID: 17130644

[23] Liu X Yuan H Niu Y Niu W Fu L . The role of AMPK/mTOR/S6K1 signaling axis in mediating the physiological process of exercise-induced insulin sensitization in skeletal muscle of C57BL/6 mice. Biochim Biophys Acta (BBA)-Molecular Basis Dis. (2012) 1822:1716–26. doi: 10.1016/j.bbadis.2012.07.008. PMID: 22846606

[24] Fan J Zhang X Zhang J Zhao T Burley SK Zheng XS . PDX1 phosphorylation at S61 by mTORC1 links nutrient signaling to β cell function and metabolic disease. Cell Rep. (2026) 45. doi: 10.1016/j.celrep.2025.116811. PMID: 41528843 PMC12949489

[25] Pedersen BK . Physical activity and muscle–brain crosstalk. Nat Rev Neurosci. (2019) 20:419–30. doi: 10.1038/s41574-019-0174-x. PMID: 30837717

[26] Letukienė A Hendrixson V Ginevičienė V . Current knowledge and scientific trends in myokines and exercise research in the context of obesity. Front Med (Lausanne). (2024) 11:1421962. 39376657 10.3389/fmed.2024.1421962 PMC11456489

[27] Pedersen L Hojman P . Muscle-to-organ cross talk mediated by myokines. Adipocyte. (2012) 1:164–7. doi: 10.4161/adip.20344. PMID: 23700527 PMC3609091

